# Combination of Eight Alleles at Four Quantitative Trait Loci Determines Grain Length in Rice

**DOI:** 10.1371/journal.pone.0150832

**Published:** 2016-03-04

**Authors:** Yuxiang Zeng, Zhijuan Ji, Zhihua Wen, Yan Liang, Changdeng Yang

**Affiliations:** State Key Laboratory of Rice Biology, China National Rice Research Institute, Hangzhou, 310006, People’s Republic of China; Nanjing Forestry University, CHINA

## Abstract

Grain length is an important quantitative trait in rice (*Oryza sativa* L.) that influences both grain yield and exterior quality. Although many quantitative trait loci (QTLs) for grain length have been identified, it is still unclear how different alleles from different QTLs regulate grain length coordinately. To explore the mechanisms of QTL combination in the determination of grain length, five mapping populations, including two F_2_ populations, an F_3_ population, an F_7_ recombinant inbred line (RIL) population, and an F_8_ RIL population, were developed from the cross between the U.S. tropical *japonica* variety ‘Lemont’ and the Chinese *indica* variety ‘Yangdao 4’ and grown under different environmental conditions. Four QTLs (*qGL-3-1*, *qGL-3-2*, *qGL-4*, and *qGL-7*) for grain length were detected using both composite interval mapping and multiple interval mapping methods in the mapping populations. In each locus, there was an allele from one parent that increased grain length and another allele from another parent that decreased it. The eight alleles in the four QTLs were analyzed to determine whether these alleles act additively across loci, and lead to a linear relationship between the predicted breeding value of QTLs and phenotype. Linear regression analysis suggested that the combination of eight alleles determined grain length. Plants carrying more grain length-increasing alleles had longer grain length than those carrying more grain length-decreasing alleles. This trend was consistent in all five mapping populations and demonstrated the regulation of grain length by the four QTLs. Thus, these QTLs are ideal resources for modifying grain length in rice.

## Introduction

Rice (*Oryza sativa* L.) is a staple food crop for more than half of the world’s population. Therefore, rice yield is the primary objective of rice breeding programmes, because food security is continuously challenged by multiple factors, including increasing population, reduced arable land, global climate change, and increasing demand for biofuel production [1]. Grain shape (or size) is a key determinant of grain yield, because it is closely correlated with grain weight, one of the three major components (grain number, panicle number, and grain weight) that determine rice yield [1, 2].

Grain shape (or size) in rice is characterized by a combination of four parameters: grain length, grain width, grain length-to-width ratio, and grain thickness [2]. These four parameters are typical polygenic traits controlled by quantitative trait loci (QTLs). Being a hot point in rice breeding programmes, grain shape has been extensively studied using various genetic-based approaches. Many QTLs for grain shape and grain weight have been mapped, and some of them have been also cloned and characterized. At least six QTLs for grain length have been fine mapped including *qGRL1* [3], *qSS7* [4], *GS7* [5], *qGL7* [6], *qGL4b* [7], and *qGL-3a* [8]; at least nine QTLs for grain weight have been fine mapped including *gw3*.*1* [9], *GW3* [10], *GW6* [10], *gw8*.*1* [11], *gw9*.*1* [12], *tgw11* [13], *GW1-1* [14], *GW1-2* [14], and *qTGW3*.*2* [15]; and a total of nine QTLs for grain size have been isolated and cloned including *GS3* [16, 17], *GS5* [18], *qGL3*/*qGL3*.*1*/*OsPPKL1* [19–21], *GW2* [22], *GW5*/*qSW5* [23, 24], *GW8* [25], *GIF1* [26], *TGW6* [27], and *qGW7/GL7* [28, 29].

The characterization of cloned QTLs suggests that multiple signaling pathways, such as ubiquitination-mediated proteasomal degradation, phytohormones, and G-protein signaling pathways, are involved in the determination of grain length [1]. For instance, *GW2* and *GW5*/*qSW5* that encode a RING-type E3 ubiquitin ligase and a nuclear protein that interacts with polyubiquitin [22–24], respectively, participate in the ubiquitination-mediated proteasomal degradation pathway; and *TGW6* that encodes a protein with indole-3-acetic-acid (IAA)-glucose hydrolase activity participates in the phytohormone pathway and regulates the level of free IAA in grains [27].

Although the underlying molecular mechanisms of grain size regulation have been elucidated in rice, information regarding the relationship between different QTLs is limited. The relationship of four QTLs for seed size (*GS3*, *GW2*, *GW5*/*qSW5*, and *GIF1*) was studied by RNA interference technology using reverse transcription polymerase chain reaction, and it was found that *GW2* and *GW5*/*qSW5* positively regulate the expression of *GS3*, and that *GIF1* is positively regulated by *GW5*/*qSW5*, but negatively regulated by *GS3* and *GW2* [30]. Rice plants carrying both *GW2* and *GW5*/*qSW5* alleles showed a significant increase in grain width compared to those carrying one of the two alleles, suggesting that *GW2* and *GW5*/*qSW5* may participate in independent pathways [31]. The development and study of the *gs3*/*gw8* double mutant in a near-isogenic background [25] showed that *GS3* and *GW8* also participate in independent pathways [1].

A large number of QTLs for grain length have been identified using various mapping populations [2]. However, the regulation of rice grain length by different alleles at the grain length-related QTLs is still poorly understood. In this study, we first developed three primary QTL mapping populations (two F_2_ and a F_3_) by crossing two rice varieties, ‘Lemont’ and ‘Yangdao 4’. These three mapping populations had been used initially to detect sheath blight resistance QTLs in a previous study [32]. QTL analysis using these primary mapping populations identified four grain length-related QTLs. Interestingly, the grain length was coordinately regulated by the eight alleles at these four QTLs. We then used two permanent mapping populations (a F_7_ and a F_8_, developed by crossing ‘Lemont’ and ‘Yangdao 4’) to further test whether the four QTLs act additively across loci, leading to a linear relationship between predicted breeding value of QTLs and phenotype, or act epistatically leading to a clearly nonlinear relationship. Using data collected in five mapping populations, we have thus demonstrated the regulation of rice grain length by the combination of eight alleles at four QTLs.

## Materials and Methods

### Mapping Populations

‘Lemont’, a U.S. tropical *japonica* variety, was crossed with ‘Yangdao 4’, a Chinese *indica* variety, to develop five mapping populations, including two F_2_ populations, an F_3_ population, an F_7_ recombinant inbred line (RIL) population, and an F_8_ RIL population. ‘Lemont’ and ‘Yangdao 4’ were provided by Dr. Xinghua Wei at China National Rice Research Institute (CNRRI).

The F_2_ populations with 190 and 182 plants each were sown in May 2011 and May 2012, respectively, in Fuyang, Hangzhou (119°95′E, 30°07′N), at the farm of CNRRI, arranged with 6 plants in each row with spacing of 17 and 20 cm between plants and between rows, respectively. The F_3_ population with 160 lines that derived from the former F_2_ population was sown in November 2012 in Lingshui, Hainan (110°02′E, 18°48′N), at the trial station of CNRRI. A total of 18 plants were grown from each of the 160 lines, arranged in three rows of six plants each. The three mapping populations were also used in a previous study to detect sheath blight resistance QTLs [32].

The F_7_ RIL population with 220 lines was sown in May 2014 in Fuyang, Hangzhou (119°95′E, 30°07′N), at the farm of CNRRI, while the F_8_ RIL population that derived from the F_7_ RIL population was sown in November 2014 in Lingshui, Hainan (110°02′E, 18°48′N), at the trial station of CNRRI. A total of 18 plants were grown from each of the 220 lines in both RIL populations, arranged in three rows of six plants each. Field management included all the common agronomic practices in Hangzhou and Hainan.

### Measurement of Grain Length

Harvested rice grains were sun-dried and stored at room temperature for at least 1 month before the measurement of grain length using a vernier caliper. Ten fully filled grains were randomly selected from the upper half of the panicle of each individual plant in the F_2_ populations and measured. Ten plants within each line of the F_3_ population were randomly selected, and five grains per plant (50 grains in total per line) were measured. In the F_7_ and F_8_ RIL population, 16 and 25 grains, respectively, were randomly selected from each line and measured. In all mapping populations, the average grain length was used for analysis.

### Molecular Marker Assays and QTL Analysis

DNA extraction and PCR were performed as described by Zhang et al. [33] and Zeng et al. [34], respectively. Briefly, a total of 179 polymorphic markers were used in QTL analysis. All the 179 markers are co-dominant markers, including simple sequence repeat and insertion-deletion markers [34]. Composite interval mapping (CIM) was used to identify QTLs for grain length using Windows QTL Cartographer 2.5 (http://statgen.ncsu.edu/qtlcart/WQTLCart.htm). The threshold of the logarithm of odds (LOD) that declares the existence of a QTL was determined based on 1,000 permutations (*P* < 0.05). Inclusive composite interval mapping (ICIM) was used to detect digenic epistatic loci using QTL IciMapping v. 4.0.6.0 [35] based on 1,000 permutations (*P* < 0.05). The digenic epistatic loci were also confirmed by two-way analysis of variance (ANOVA).

A fast and simple method based on CIM was used to identify QTLs for grain length in the five mapping populations. First, a genome-wide linkage map that was constructed using 179 polymorphic markers [32] was used to identify QTLs for grain length in the F_2_ population grown in 2011 in Hangzhou, and in the F_3_ population grown in 2012 in Hainan; this revealed three QTLs. Second, the three detected QTLs for grain length were further examined in the F_2_ population grown in 2012 in Hangzhou, based on a linkage map that was constructed using 44 markers (S1 Table). These 44 markers covered the regions of the three QTLs for grain length that were detected in the F_2_ population grown in 2011 in Hangzhou, and in the F_3_ population, and represented a total of 443.3 cM with an average of 13.4 cM between adjacent markers. The three QTLs were not repeatedly detected, but a new QTL was identified, yielding a total of four QTLs in the three primary mapping populations. Third, the four QTLs for grain length detected in the two F_2_ and the F_3_ populations were further examined in the F_7_ and F_8_ RIL populations using 21 markers (S1 Fig). The 21 markers covered the regions of all the four QTLs for grain length that were detected in the two F_2_ and the F_3_ populations, and represented a total of 112.1 cM with an average of 6.6 cM between adjacent markers.

Since the multiple interval mapping (MIM) method may provide greater power and precision for QTL mapping [36], we used it to detect QTLs, and compared it with the CIM method in the five mapping populations using Windows QTL Cartographer 2.5. In all the mapping populations, we used the following procedure to build a MIM model for QTL analysis based on the instructions provided by Silva et al. [37]. First, the MIM forward search method was used to create an initial MIM model because it is more powerful than the other options. The Bayesian information criterion (BIC)-based model selection criteria (BIC-M0 in the software) was used, with a MIM search walk speed of 1 cM. Second, several MIM model refinement rounds was performed to build a final MIM model. The model refinement rounds consisted of (1) searching repeatedly for QTLs with main effects until no main effect QTL were found, (2) searching repeatedly for pairs of epistatic QTLs until no further epistatic QTLs were found, (3) testing epistasis effects and excluding all the epistatic QTLs that were not statistically significant, (4) testing for the main effects of QTLs and excluding all the main effect QTLs that were not statistically significant, and (5) optimizing positions of both main and epistatic QTLs.

### Statistical Analysis

All analyses, including Shapiro-Wilk test, ANOVA, two-way ANOVA, and linear regression analysis, were performed using SAS v. 8.01 (SAS Institute, Cary, NC, USA).

## Results

### Identification and Confirmation of QTLs for Grain Length

**Fig 1:**
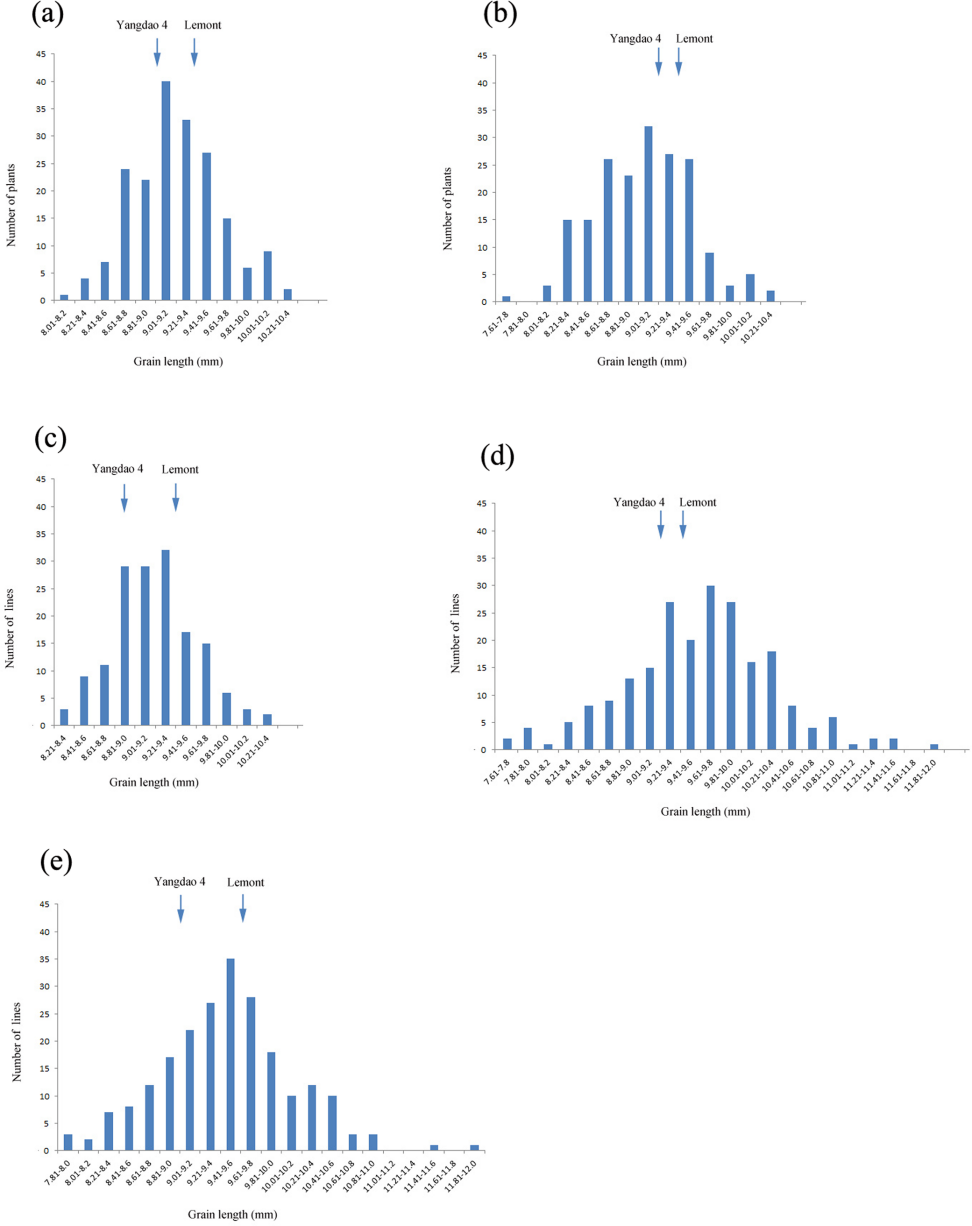
Frequency distribution of grain length in five mapping populations (two F_2_ populations, an F_3_ population, an F_7_ recombinant inbred line [RIL] population, and an F_8_ RIL population) derived from the cross between the *japonica* variety ‘Lemont’ and the *indica* variety ‘Yangdao 4’ and grown under different environmental conditions. (a) F_2_ population grown in 2011 in Hangzhou, (b) F_2_ population grown in 2012 in Hangzhou, (c) F_3_ population grown in 2012 in Hainan, (d) F_7_ recombinant inbred line (RIL) population grown in 2014 in Hangzhou, and (e) F_8_ RIL population grown in 2014 in Hainan.

The Shapiro-Wilk test was performed to determine whether the phenotypic data were normally distributed (F_2_ population grown in 2011 in Hangzhou, W = 0.98, *P* = 0.04; F_2_ population grown in 2012 in Hangzhou, W = 0.99, *P* = 0.36; F_3_ population, W = 0.99, *P* = 0.58; F_7_ recombinant RIL population, W = 0.99, *P* = 0.32; and F_8_ RIL population, W = 0.99, *P* = 0.41). The apparent normal distribution of grain length in four of the five mapping populations indicated that grain length was a quantitative trait controlled by polygenes (Fig 1).

Three QTLs (*qGL-3-1*, *qGL-3-2*, and *qGL-4*) were detected by CIM (Table 1, S2 Fig), using the F_2_ population grown in 2011 in Hangzhou, while no QTLs were detected using the F_3_ population. None of the three QTLs were repeatedly detected in the F_2_ population grown in 2012 in Hangzhou; however, a new QTL, *qGL-7*, was identified (Table 1, S3 Fig). The putative alleles from Yangdao 4 increased grain length at *qGL-3-1* and *qGL-4*, while in comparison the alleles from Lemont decreased the grain length. The putative alleles from Lemont increased grain length at *qGL-3-2* and *qGL-7*, whereas a relative decrease was observed with the alleles from Yangdao 4 (Table 1).

**Table 1:** Quantitative trait loci (QTLs) for grain length detected in four mapping populations (two F_2_ populations, an F_7_ recombinant inbred line [RIL] population, and an F_8_ RIL population) derived from the cross between the *japonica* variety ‘Lemont’ and the *indica* variety ‘Yangdao 4’ and grown under different environmental conditions (in 2011 in Hangzhou, in 2012 in Hangzhou, in 2014 in Hangzhou, and in 2014 in Hainan, respectively) using composite interval mapping. No QTLs were detected in the F_3_ population grown in 2012 in Hainan.

Year/Location	Mapping population	QTL	Chromosome	LOD	Marker interval^a^/Physical position (Mb)^b^	Nearest marker	R^2^%	Additive effect (mm)	Dominance effect	Direction of phenotypic effect^c^
2011/Hangzhou	F_2_	*qGL-3-1*	3	5.01	D307-D311/6.1–10.1	D309	11.29	-0.20	0.04	YD
		*qGL-3-2*	3	6.23	D336B-RM3585/36.1–37.0	RM3585	10.83	0.20	0.00	LE
		*qGL-4*	4	8.29	D456-RM1113/30.3–34.7	D463	12.64	-0.24	-0.05	YD
2012/Hangzhou	F_2_	*qGL-7*	7	3.59	RM234-D755/26.1–27.5	D755	2.24	0.47	0.31	LE
2014/Hangzhou	F_7_ RIL	*qGL-3-2*	3	4.17	D334-RM3585/33.4–37.0	D336B	8.66	0.29	-	LE
		*qGL-4*	4	4.27	D460A- D467/32.7–35.7	D463	7.71	-0.27	-	YD
		*qGL-7*	7	2.47	D750-D754/24.9–27.0	RM234	4.46	0.22	-	LE
2014/Hainan	F_8_ RIL	*qGL-3-2*	3	2.08	D336B-RM3585/36.1–37.0	D336B	5.47	0.21	-	LE
		*qGL-4*	4	4.12	D460A- D467/32.7–35.7	D463	7.81	-0.26	-	YD
		*qGL-7*	7	2.44	RM234-D754/26.1–27.0	RM21997	4.64	0.24	-	LE

^a^Marker interval is the flanking marker that is closest to the LOD peak.

^b^Physical position of the corresponding marker as determined by BLAST tool on the National Center for Biotechnology Information (NCBI) website (www.ncbi.nlm.nih.gov) against the Nipponbare reference sequence.

^c^LE and YD denote Lemont and Yangdao 4 alleles, respectively, that increase the phenotypic value.

We used two RIL populations to further confirm the four QTLs (*qGL-3-1*, *qGL-3-2*, *qGL-4*, and *qGL-7*) detected in the three primary mapping populations. Three of the four detected QTLs (*qGL-3-2*, *qGL-4*, and *qGL-7*) were consistently identified by CIM in the F_7_ and F_8_ RIL populations (Table 1, S4 Fig). Although *qGL-3-1* was not detected by CIM, the LOD score curve from marker D309 showed a peak at the *qGL-3-1* region in both RIL populations (S4A Fig).

The MIM method then compared with the CIM method. We found that MIM was more sensitive than CIM in detecting QTL; this is shown in Table 2, and indicates that more QTLs were identified using MIM. Neither method detected any QTL in the F_3_ population planted in 2012 in Hainan. All the four QTLs identified using CIM were also detected using MIM (Table 1, Table 2). The four QTLs detected using CIM (i.e. *qGL-3-1*, *qGL-3-2*, *qGL-4*, *qGL-7*), corresponded to the four QTLs detected using MIM (i.e. *qGL-3-1MIM*, *qGL-3-2MIM*, *qGL-4MIM*, *qGL-7-2MIM*), respectively (Table 1, Table 2). The ‘MIM’ followed after the QTL name denoted QTL detected using MIM, and was used to differentiate these QTLs from those detected using CIM. The nearest markers to the four QTLs detected using CIM or MIM were the same except for one QTL, *qGL-7* or the corresponding *qGL-7-2MIM*, which was detected in the F_2_ population grown in 2012 in Hangzhou. The nearest marker to *qGL-7* was D755, while the nearest marker to *qGL-7-2MIM* was RM234 (Table 1, Table 2). The physical distance between D755 and RM234 is about 1,400 kb according to the Nipponbare reference sequence. *qGL-7* and *qGL-7-2MIM* were both located at the D755-RM234 interval; therefore, *qGL-7* and *qGL-7-2MIM* were considered to represent the same QTL. It indicated that the CIM results were consistent with the MIM results, but MIM was more sensitive than CIM. The following analysis focused on the four QTLs detected using both CIM and MIM, i.e. *qGL-3-1MIM*, *qGL-3-2MIM*, *qGL-4MIM*, and *qGL-7-2MIM*.

**Table 2:** Quantitative trait loci (QTLs) for grain length detected in four mapping populations (two F_2_ populations, an F_7_ recombinant inbred line [RIL] population, and an F_8_ RIL population) derived from the cross between the *japonica* variety ‘Lemont’ and the *indica* variety ‘Yangdao 4’ and grown under different environmental conditions (in 2011 in Hangzhou, in 2012 in Hangzhou, in 2014 in Hangzhou, and in 2014 in Hainan, respectively) using multiple interval mapping. No QTLs were detected in the F_3_ population grown in 2012 in Hainan.

Year/Location	Mapping population	QTL^a^	Chromosome	LOD	Marker interval^b^	Nearest marker	R^2^%	Additive effect (mm)	Dominance effect	Direction of phenotypic effect^c^
2011/Hangzhou	F_2_	*qGL-1MIM*	1	4.67	RM1-D108C	RM1	6.57	0.16	-0.02	LE
		*qGL-3-1MIM*	3	6.44	D307-D309	D309	9.40	-0.20	-0.04	YD
		*qGL-3-2MIM*	3	10.93	D336B-RM3585	RM3585	16.00	0.24	0.02	LE
		*qGL-4MIM*	4	12.12	D456-D463	D463	19.45	-0.27	-0.07	YD
		*qGL-7-1MIM*	7	3.8	D701-RM3831	D701	4.92	0.13	0.02	LE
		*qGL-7-2MIM*	7	4.27	RM234-D755	D755	5.86	0.14	-0.01	LE
		*qGL-8MIM*	8	3.29	D856-D860	D860	3.89	-0.11	-0.05	YD
		*qGL-10MIM*	10	2.61	D1042-D1048	D1048	3.74	-0.12	-0.06	YD
2012/Hangzhou	F_2_	*qGL-3-2MIM*	3	2.42	RM3585-D336B	RM3585	5.22	0.40	-0.05	LE
		*qGL-4MIM*	4	1.27	D456-D468	D468	2.68	-0.28	-0.12	YD
		*qGL-7-2MIM*	7	3.48	RM234-D755	RM234	7.71	0.46	0.30	LE
2014/Hangzhou	F_7_ RIL	*qGL-3-2MIM*	3	4.09	D335C-D336B	D336B	8.57	0.29	-	LE
		*qGL-4MIM*	4	4.61	RM1113-D460A	D463	8.33	-0.28	-	YD
		*qGL-7-2MIM*	7	2.57	RM505-RM21976	RM234	4.57	0.21	-	LE
2014/Hainan	F_8_ RIL	*qGL-3-2MIM*	3	2.02	D336B-RM3585	D336B	5.31	0.21	-	LE
		*qGL-4MIM*	4	4.46	RM1113-D460A	D463	8.54	-0.26	-	YD
		*qGL-7-2MIM*	7	1.7	RM21985-D754	RM21997	3.20	0.17	-	LE

^a^‘MIM’ followed after the QTL name is used to differentiate the QTLs detected by CIM.

^b^Marker interval is the flanking marker that is closest to the LOD peak.

^c^LE and YD denote Lemont and Yangdao 4 alleles, respectively, that increase the phenotypic values.

### QTL-By-Environment and QTL-By-Population Interactions

Two-way ANOVA was conducted to examine whether the four grain length-QTLs had significant interaction with the environments or populations. The markers closest to the four QTLs were used to represent the QTL genotypes at the specific populations. The heterozygotes in F_2_ populations were omitted, and were not used in analysis.

Among the four grain length-QTLs, some QTLs were not detected in some of the mapping populations (Table 1, Table 2). We determined the most likely marker to represent the QTL for those populations by analyzing only the LOD score curves in CIM results, because these results were consistent with those from MIM analysis. For *qGL-3-1* (or *qGL-3-1MIM*) that was detected only in the F_2_ population grown in 2011 in Hangzhou using either CIM or MIM, D309 was the closest marker (Table 1, Table 2, S2A Fig). Although this QTL was not detected in the F_7_ and F_8_ RIL populations by CIM or MIM, the LOD score curve from D309 showed a peak (S4A Fig) at this QTL region. Therefore, the D309 genotype was used to represent the *qGL-3-1* genotype for the other four mapping populations. For *qGL-3-2* (or *qGL-3-2MIM*) that was detected in four populations using MIM, both D336B and RM3585 were the closest markers (Table 1, Table 2). We chose D336B as the closest marker to this QTL for the F_3_ population planted in 2012 in Hainan, because D336B was the most proximal marker in the F_7_ and F_8_ RIL populations analyzed using CIM or MIM, and RIL populations are generally considered more reliable than F_2_ populations. For *qGL-4* (or *qGL-4MIM*) that was detected in four populations using MIM, D463 was the closest marker to this QTL in three populations (Table 2).Therefore, D463 was chosen as the closest marker to this QTL for the F_3_ population planted in 2012 in Hainan. The closest markers for *qGL-7* (or *qGL-7-2MIM*) were differed according to the mapping populations derived from CIM or MIM analysis (Table 1, Table 2). To identify the most suitable marker, the LOD score curves in the F_7_ and F_8_ RIL populations were analyzed. Two peaks were observed in the RIL populations, that is, one peak in the F_7_ RIL population and another peak in the F_8_ RIL population, which were co-located at the RM234 position (S4D Fig). Therefore, the RM234 genotype was used to represent the *qGL-7* genotype for the F_3_ population planted in 2012 in Hainan.

ANOVA showed that there was no significant interaction between each QTL and the environment (Table 3). Significant interaction was not detected between each QTL and mapping population (Table 4).

**Table 3:** Two-way ANOVA used to detect QTL-by-environment interaction. In different mapping populations, the closest Markers to the four grain length-QTLs (*qGL-3-1MIM*, *qGL-3-2MIM*, *qGL-4MIM*, and *qGL-7-2MIM*) were used to represent the QTL genotypes at the specific populations. Since there were five mapping environments, the degree of freedom for environment is four. There were two genotypes at each QTL (the heterozygotes were not used in analysis), and the degree of freedom for QTL is 1.

QTL by environment interaction	Degree of freedom	Type I sum of squares	Mean square	F value	*P*
Environment	4	18.29	4.57	11.98	<0.01
QTL	1	7.65	7.64	20.04	<0.01
*qGL-3-1MIM* × Environment	4	0.55	0.14	0.36	0.84
Environment	4	20.12	5.03	14.51	<0.01
QTL	1	27.24	27.24	78.61	<0.01
*qGL-3-2MIM* × Environment	4	0.90	0.22	0.65	0.63
Environment	4	23.75	5.94	18.36	<0.01
QTL	1	26.39	26.39	81.64	<0.01
*qGL-4MIM* × Environment	4	0.36	0.09	0.28	0.89
Environment	4	24.02	6.01	17.81	<0.01
QTL	1	21.60	21.60	64.06	<0.01
*qGL-7-2MIM* × Environment	4	0.71	0.18	0.53	0.72

**Table 4:** Two-way ANOVA used to detect QTL-by-population interaction. In different mapping populations, the closest Markers to the four grain length-QTLs (*qGL-3-1MIM*, *qGL-3-2MIM*, *qGL-4MIM*, and *qGL-7-2MIM*) were used to represent the QTL genotypes at the specific populations. Since there were three types of mapping populations (F_2_, F_3_, and RIL), the degree of freedom for population is two. There were two genotypes at each QTL (the heterozygotes were not used in analysis), and the degree of freedom for QTL is 1.

QTL by population interaction	Degree of freedom	Type I sum of squares	Mean square	F value	*P*
Population	2	14.71	7.35	19.27	<0.01
QTL	1	8.11	8.11	21.25	<0.01
*qGL-3-1MIM* × Population	2	0.19	0.09	0.24	0.78
Population	2	17.59	8.80	25.16	<0.01
QTL	1	27.21	27.21	77.83	<0.01
*qGL-3-2MIM* × Population	2	0.06	0.03	0.08	0.92
Population	2	19.32	9.66	29.54	<0.01
QTL	1	27.39	27.39	83.80	<0.01
*qGL-4MIM* × Population	2	0.07	0.03	0.10	0.90
Population	2	20.62	10.31	30.16	<0.01
QTL	1	21.28	21.28	62.26	<0.01
*qGL-7-2MIM* × Population	2	0.03	0.02	0.05	0.95

### Digenic Epistasis in Five Mapping Populations

ICIM was used to identify epistatic loci for grain length across all 12 chromosomes (S2 Table); these loci were further confirmed by two-way ANOVA (S3 Table). Four pairs of digenic epistatic loci were detected in the F_2_ population grown in 2011 in Hangzhou (S5 Fig), while no digenic epistatic loci were detected in the F_3_ population and the F_2_ population grown in 2012 in Hangzhou. Digenic epistatic loci were not detected in RIL mapping populations, probably due to the low number of markers used for genotyping.

MIM detected only a pair of epistatic QTLs in the F_2_ population grown in 2011 in Hangzhou, i.e., *qGL-4MIM* by *qGL-7-1MIM* interaction, which explained 1.93% of the epistatic variation, and the dominance-by-dominance effect was -0.24. MIM did not detect other epistatic loci in the other four mapping populations. Overall, these results show that epistasis does play a role in the regulation of grain length.

### The Four Grain-Length-QTLs Act Additively across Loci in Regulation of Grain Length

Digenic epistasis was not detected between the four grain-length-QTLs in all the five mapping populations, indicating that the four QTLs acted predominantly in an additive manner. The additive effects of the QTLs were similar within each mapping population (Table 1), revealing that the four QTLs might have similar effects on the regulation of grain length.

In each of the four loci (*qGL-3-1*, *qGL-3-2*, *qGL-4*, and *qGL-7*), there was an allele from one parent that increased grain length and another allele from another parent that decreased it. The four grain length-increasing alleles were *qGL-3-1YD*, *qGL-3-2LE*, *qGL-4YD*, and *qGL-7LE*, respectively, in the four loci. The four grain length-decreasing alleles were *qGL-3-1LE*, *qGL-3-2YD*, *qGL-4LE*, and *qGL-7YD*, respectively, in the four loci. The ‘*LE*’ or ‘*YD*’ suffix that follows the QTL name indicates whether the putative allele was from Lemont or Yangdao 4, respectively. To determine whether the eight alleles in the four QTLs act additively across loci, and lead to a linear relationship between predicted breeding values of QTLs and phenotype, linear regression analysis was performed.

First, the genotypic value of each plant in five mapping populations was calculated based on genotypes of the four QTLs, and was used as the predicted breeding value of the four QTLs. Then, linear regression analysis between the genotypic value and the grain length of all the individual plants in the five populations was performed. The genotypic value of each plant was determined by adding up the estimated additive effects of each of the four QTLs in the RIL populations, or by adding up the estimated additive effects and dominance effects of the four QTLs in the F_2_ populations. The dominance effects of the heterozygotes were used and summed up with the additive effects of the homozygotes across four loci in the F_2_ populations. When calculating genotypic values, the positive additive effect was used if a locus carried a grain length-increasing allele, and the negative additive effect was used if a locus carried a grain length-decreasing allele. The estimated additive effects or dominance effects of the four putative QTLs in the five mapping populations were determined by the MIM method using Windows QTL Cartographer 2.5.

**Fig 2:**
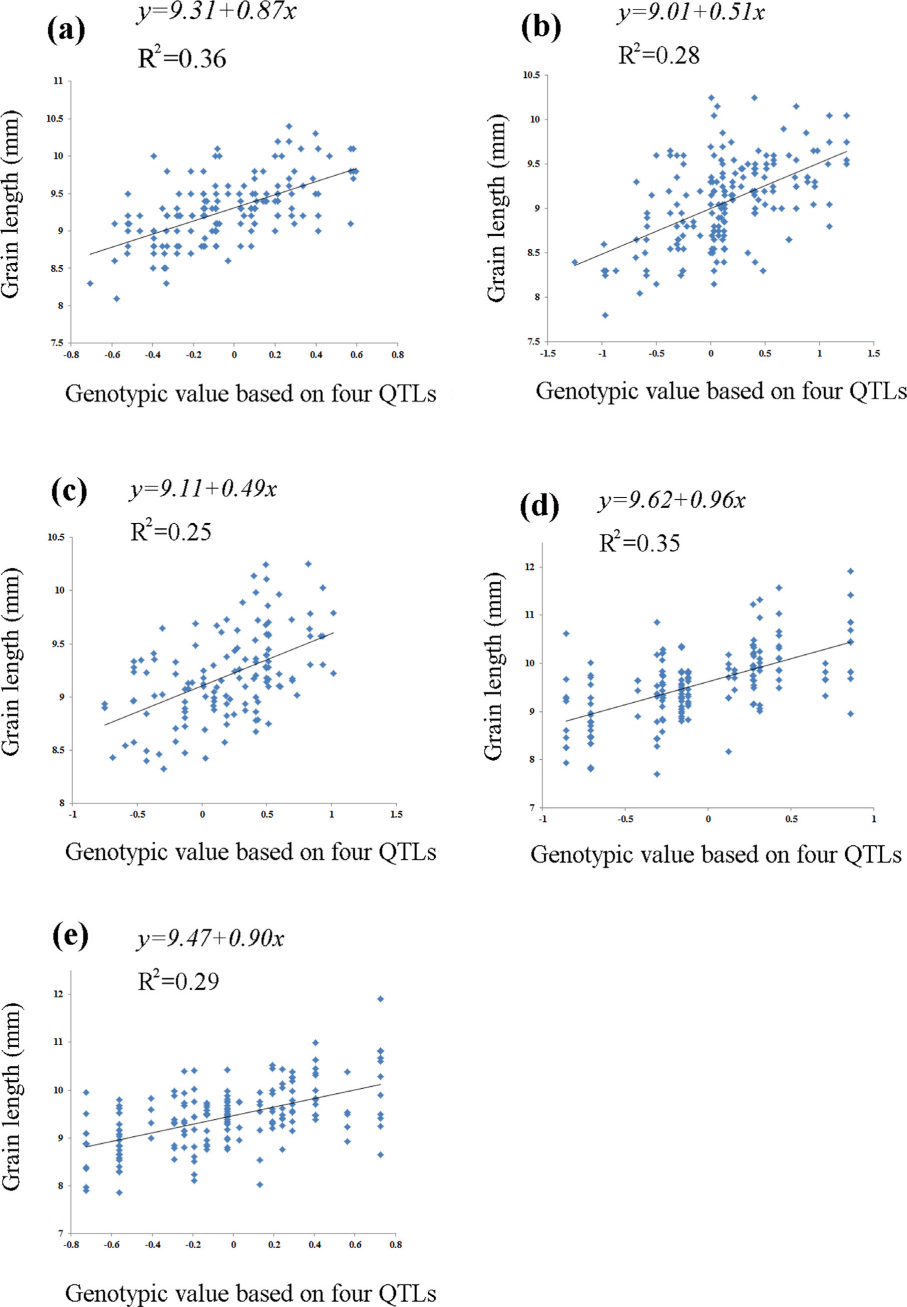
Linear regression analysis between grain length and genotypic value of individual plants in five mapping populations (two F_2_ populations, an F_3_ population, an F_7_ recombinant inbred line [RIL] population, and an F_8_ RIL population) derived from the cross between the *japonica* variety ‘Lemont’ and the *indica* variety ‘Yangdao 4’ and grown under different environmental conditions. (a) F_2_ population grown in 2011 in Hangzhou, (b) F_2_ population grown in 2012 in Hangzhou, (c) F_3_ population grown in 2012 in Hainan, (d) F_7_ RIL population grown in 2014 in Hangzhou, and (e) F_8_ RIL population grown in 2014 in Hainan. The genotypic value of an individual plant was calculated by adding up the estimated additive effects of all the four QTLs in the RIL populations, or by adding up the estimated additive effects and dominance effects of the four QTLs in the F_2_ populations. The dominance effects of the heterozygotes were used and summed up with the additive effects of the homozygotes across four loci to determine genotypic values in the F_2_ populations. When calculating genotypic values, the positive additive effect was used if a locus carried a grain length-increasing allele, and the negative additive effect was used if a locus carried a grain length-decreasing allele.

The linear regression analysis indicated a clear linear relationship between predicted breeding value and phenotype, and yielded five regression equations in the five populations (Fig 2; F_2_ population grown in 2011 in Hangzhou, F = 86.39, *P* < 0.0001; F_2_ population grown in 2012 in Hangzhou, F = 71.04, *P* < 0.0001; F_3_ population, F = 42.39, *P* < 0.0001; F_7_ recombinant RIL population, F = 91.53, *P* < 0.0001; and F_8_ RIL population, F = 70.46, *P* < 0.0001). The coefficient of determination (R^2^) was used as an estimate of the cumulative heritability of the four QTLs. The cumulative heritability of the four QTL genotypes was 36%, 28%, 25%, 35%, and 29%, respectively, in the five mapping populations. The results shown in Fig 2 suggest that the grain length was coordinately regulated by the eight alleles in the four QTLs. Plants carrying more grain length-increasing alleles had longer grain length than those carrying more grain length-decreasing alleles.

## Discussion

### Regulation of Grain Length by Eight Alleles at Four QTLs in Five Mapping Populations

Grain length is one of the most important agronomic traits in rice breeding and production [1, 2, 38] because it is positively correlated with grain weight, and influences both rice yield and other market values [2]. Long, slender grains are generally preferred by consumers in southern China, the USA, and other Southern or Southeast Asian countries; however, consumers in Japan, Korea, and northern China prefer short, round rice grains [2].

In the present study, we evaluated and analyzed five mapping populations grown under different environmental conditions and found that grain length was regulated by at least four QTLs: *qGL-3-1*, *qGL-3-2*, *qGL-4*, and *qGL-7*. Regression analysis revealed that the eight alleles at the four QTLs act additively in the regulation of grain length, leading to a linear relationship between predicted breeding value and phenotype. These results were consistent in all five mapping populations, demonstrating the regulation of grain length by the four QTLs (Fig 2).

### Stability of Four QTLs in Five Mapping Populations

QTL analysis is a genetic research approach that can reveal the underlying genetic mechanisms controlling the agronomic traits [39]. However, QTLs, particularly those with minor effects, are easily affected by environmental factors [40]. In this study, it seemed that *qGL-3-1* was hard to detect, because it was only detected in the F_2_ population grown in 2011 in Hangzhou using both CIM and MIM method, while the other three QTLs (*qGL-3-2*, *qGL-4*, and *qGL-7*) were more easily identified (Table 1, Table 2). However, significant QTL-by-environment interaction was not detected (Table 3); this was also the case regarding QTL-by-population interaction (Table 4). Although *qGL-3-1* was not detected easily by CIM or MIM, its effect on grain length was stable in all mapping populations and environments (Fig 2).

### Comparison of the Rice QTLs Associated with Grain Shape between Present and Previous Studies

**Fig 3:**
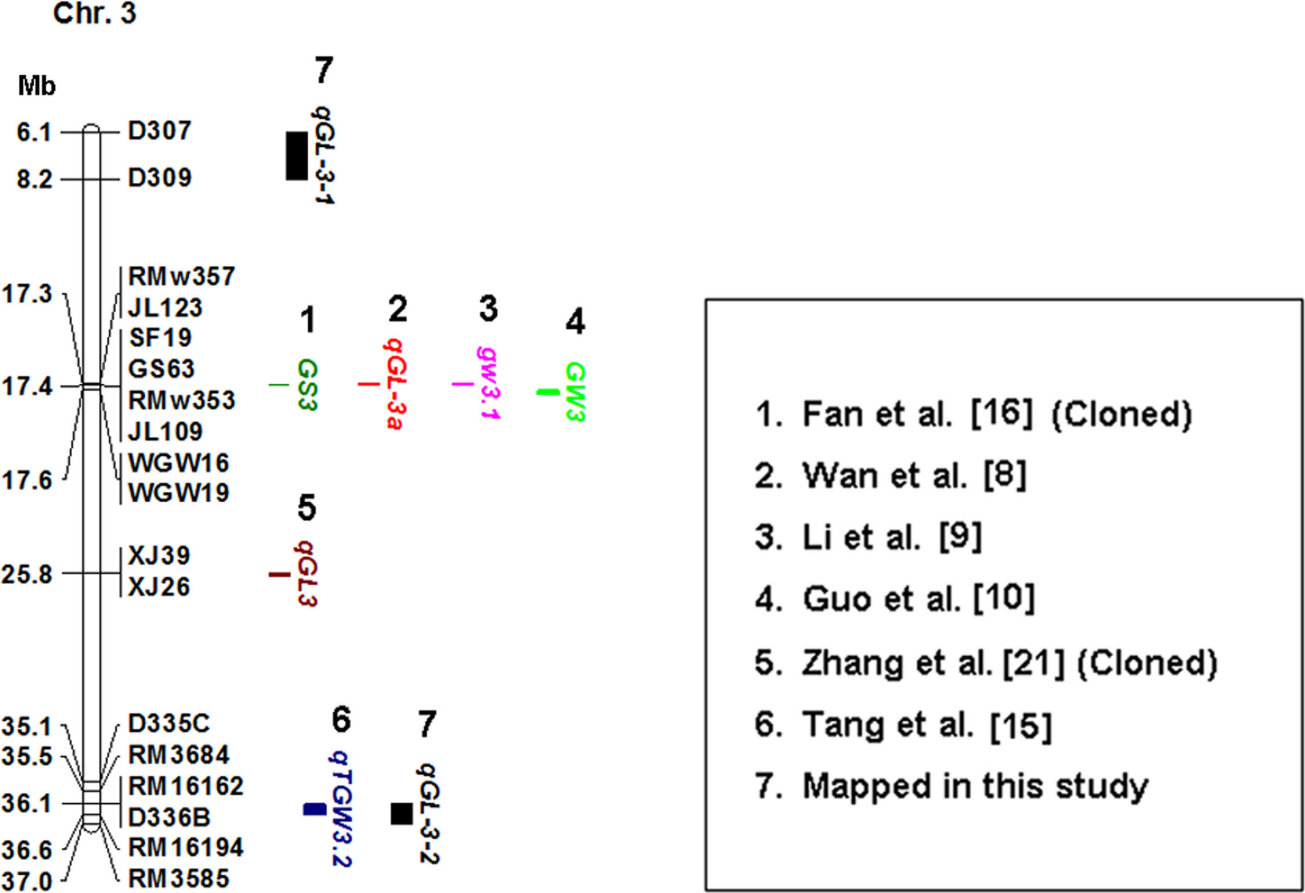
Comparison of the grain shape-related genes mapped on chromosome 3 in present and previous studies. 1. Fan et al. [16]; 2. Wan et el. [8]; 3. Li et al. [9]; 4. Guo et al. [10]; 5. Zhang et al. [21]; 6. Tang et al. [15]; 7. QTL mapped in this study. Numbers to the left of the chromosome bar indicate physical position (Mb) of the corresponding markers.

**Fig 4:**
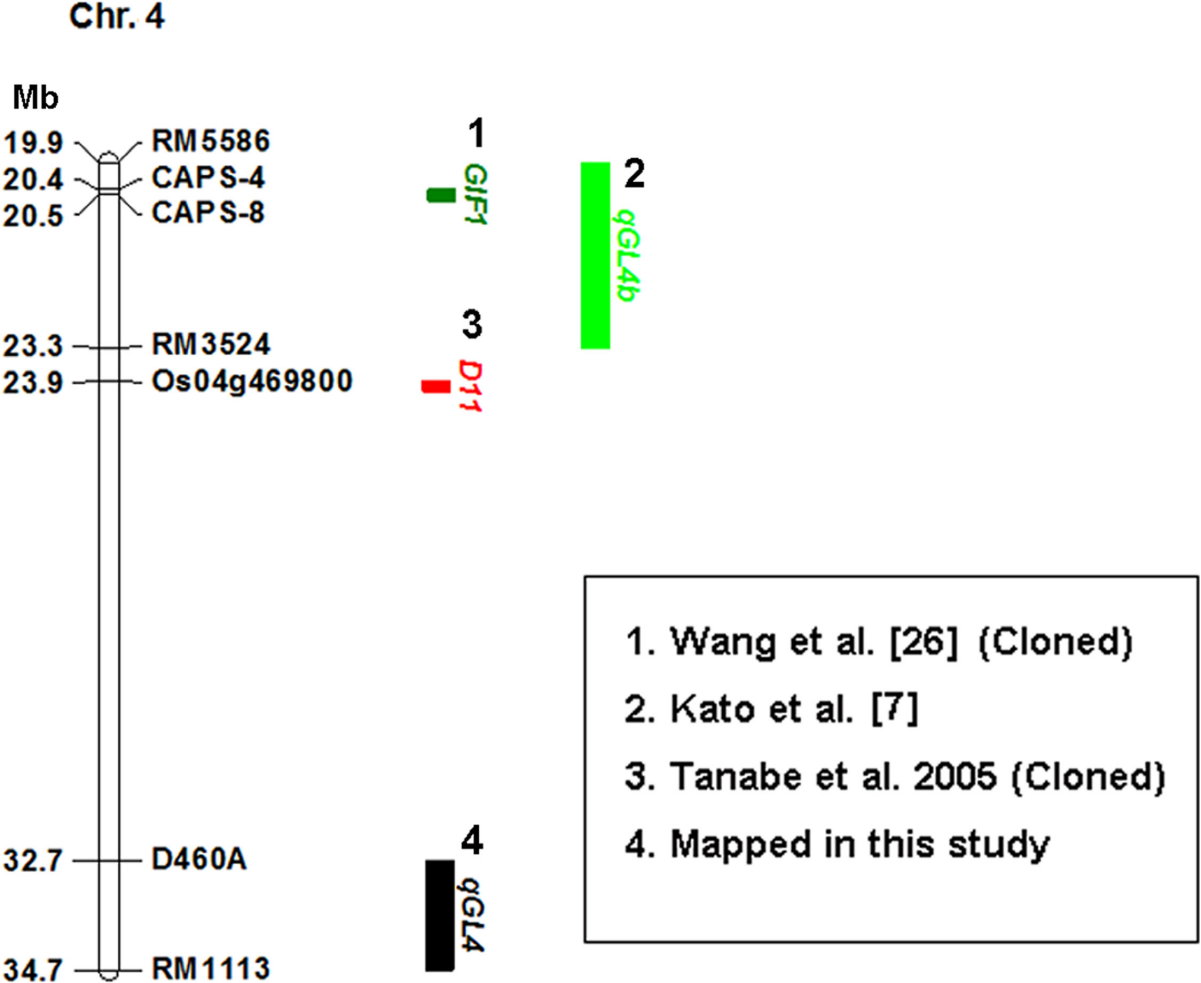
Comparison of the grain shape-related genes mapped on chromosome 4 in present and previous studies. 1. Wang et al. [26]; 2. Kato et al. [7]; 3. Tanabe et al. Plant Cell. 2005; 17:776–790; 4. QTL mapped in this study. Numbers to the left of the chromosome bar indicate physical position (Mb) of the corresponding markers.

**Fig 5:**
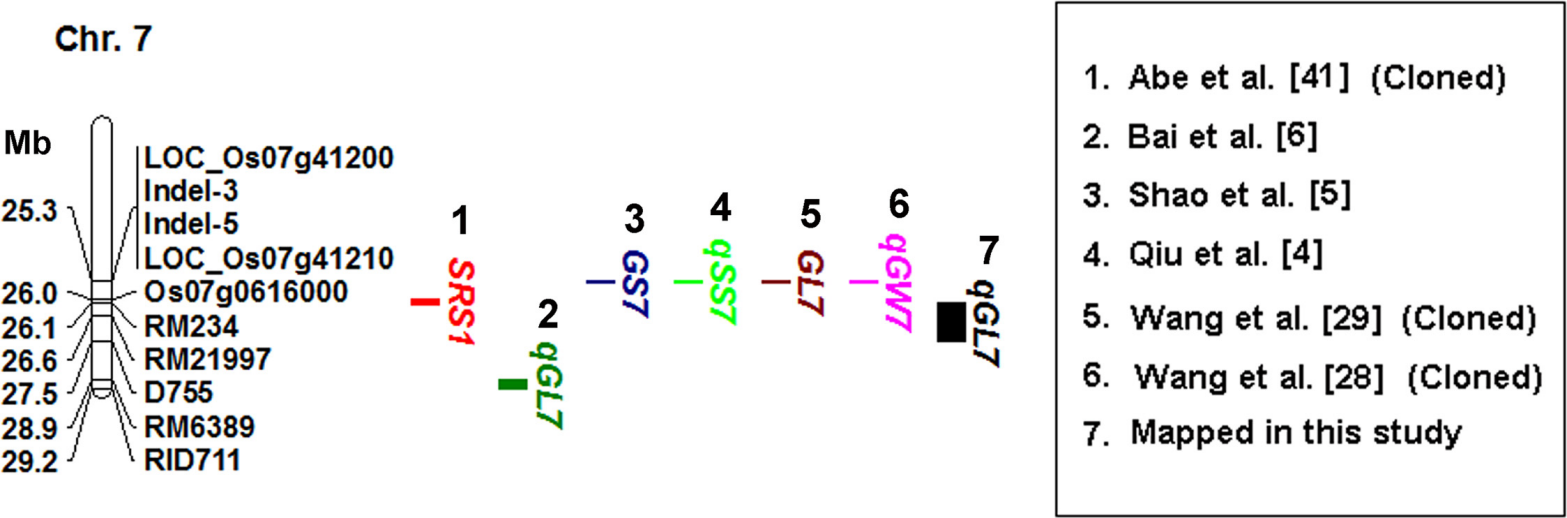
Comparison of the grain shape-related genes mapped on chromosome 7 in present and previous studies. 1. Abe et al. [41]; 2. Shao et al. [5]; 3. Qiu et al. [4]; 4. Bai et al. [6]; 5. Wang et al. [29]; 6. Wang et al. [28]; 7. QTL mapped in this study. Numbers to the left of the chromosome bar indicate physical position (Mb) of the corresponding markers.

The grain shape-related QTLs that have been fine-mapped or validated on chromosome 3, chromosome 4, and chromosome 7 have been listed in three physical maps, and compared with the grain length-QTLs identified in this study (Figs 3–5). The marker intervals of the four grain length-QTL were determined according to the MIM results (Table 2). The marker intervals for the four grain length-QTLs were D307-D309, D336B-RM3585, D460A-RM1113, and RM234-D755, respectively. We did not find any grain shape-related QTLs that had been fine-mapped at the *qGL-3-1* or the *qGL-4* region (Fig 3, Fig 4). The *qGL-3-2* was co-localized with a thousand-grain weight QTL, *qTGW3*.*2*, which was reported by Tang et al. [15]. However, it is not clear whether *qGL-3-2* and *qTGW3*.*2* are allelic based on available information. The *qGL-7* was co-located with a grain shape-related gene, *SRS1* [41]. And it was close to four grain shape-related genes, including *GS7* [5], *qSS7* [4], *qGW7* [28] and *GL7* [29] (Fig 5), The *SRS1*, *GL7* and *qGW7* have been cloned. *GL7* and *qGW7* are allelic, and both correspond to the *LOC_Os07g41200* gene, which encodes a TONNEAU1–recruiting motif protein [28, 29].

### Epistasis Influencing Rice Grain Length

Epistasis or interactions between non-allelic genes play complex roles in the control of quantitative traits in plants [42]. However, it is difficult to identify the complete epistatic networks, because most of the available software for mapping epistatic loci only detects digenic epistatic loci. Epistatic effects have rarely been observed for grain length [2]. In this study, digenic epistasis did play a role in the determination of grain length, although the four QTLs did not interact with each other. It suggested that the genetic regulation network for grain length is complicated; and the regulation of grain length by eight alleles at the four QTLs revealed only part of the regulatory mechanisms.

The additive phenotypes of the four QTLs indicated that these four loci did not act in a simple linear pathway. Since an interaction between these four loci was not found, we infer that they may participate in four distinct genetic pathways.

### Development of Cultivars with Different Grain Length by Pyramiding QTLs Carrying Suitable Alleles

The four grain length-increasing alleles and the four grain length-decreasing alleles found in the present study are ideal resources for modifying grain length in rice. Marker-assisted selection using the nearest markers to these QTLs may be applied for developing new rice cultivars with longer or shorter grain length by pyramiding different number of grain length-increasing or decreasing alleles. Breeding by design [43] using these four QTLs is still not applicable due to the limited understanding of the genetic regulation network, because it is still unclear whether other genes interact with these four QTLs. Further study is needed to investigate whether the four QTLs have the same pyramiding effect when introduced into cultivars with different genetic backgrounds.

## Supporting Information

S1 Fig Marker linkage map used for detecting QTLs in an F_7_ RIL population and an F_8_ RIL population. (DOCX)

S2 Fig LOD score curves of QTLs for grain length detected in an F_2_ population grown in 2011 in Hangzhou. (DOCX)

S3 Fig LOD score curves of QTLs for grain length detected in an F_2_ population grown in 2012 in Hangzhou. (DOCX)

S4 Fig LOD score curves of QTLs for grain length detected in an F_7_ RIL population and an F_8_ RIL population grown in 2014 in Hangzhou and Hainan, respectively. (DOCX)

S5 Fig Digenic epistatic loci detected in an F_2_ population grown in 2011 in Hangzhou. (DOCX)

S1 Table Markers used to construct a linkage map that was used for detecting QTLs in an F_2_ population grown in 2012 in Hangzhou. (DOCX)

S2 Table Pairs of digenic epistatic loci detected in an F_2_ population grown in 2011 in Hangzhou. (DOCX)

S3 Table Two-way ANOVA used to confirm the digenic epistatic loci detected in an F_2_ population grown in 2011 in Hangzhou. (DOCX)
