# Supplementary material for: Combination of Eight Alleles at Four Quantitative Trait Loci Determines Grain Length in Rice
Source: PLoS One. 2016 Mar 4;11(3):e0150832. doi: 10.1371/journal.pone.0150832 (PMC4778864; doi:10.1371/journal.pone.0150832)
Supplement: S4 Fig — (DOCX) [file pone.0150832.s004.docx]

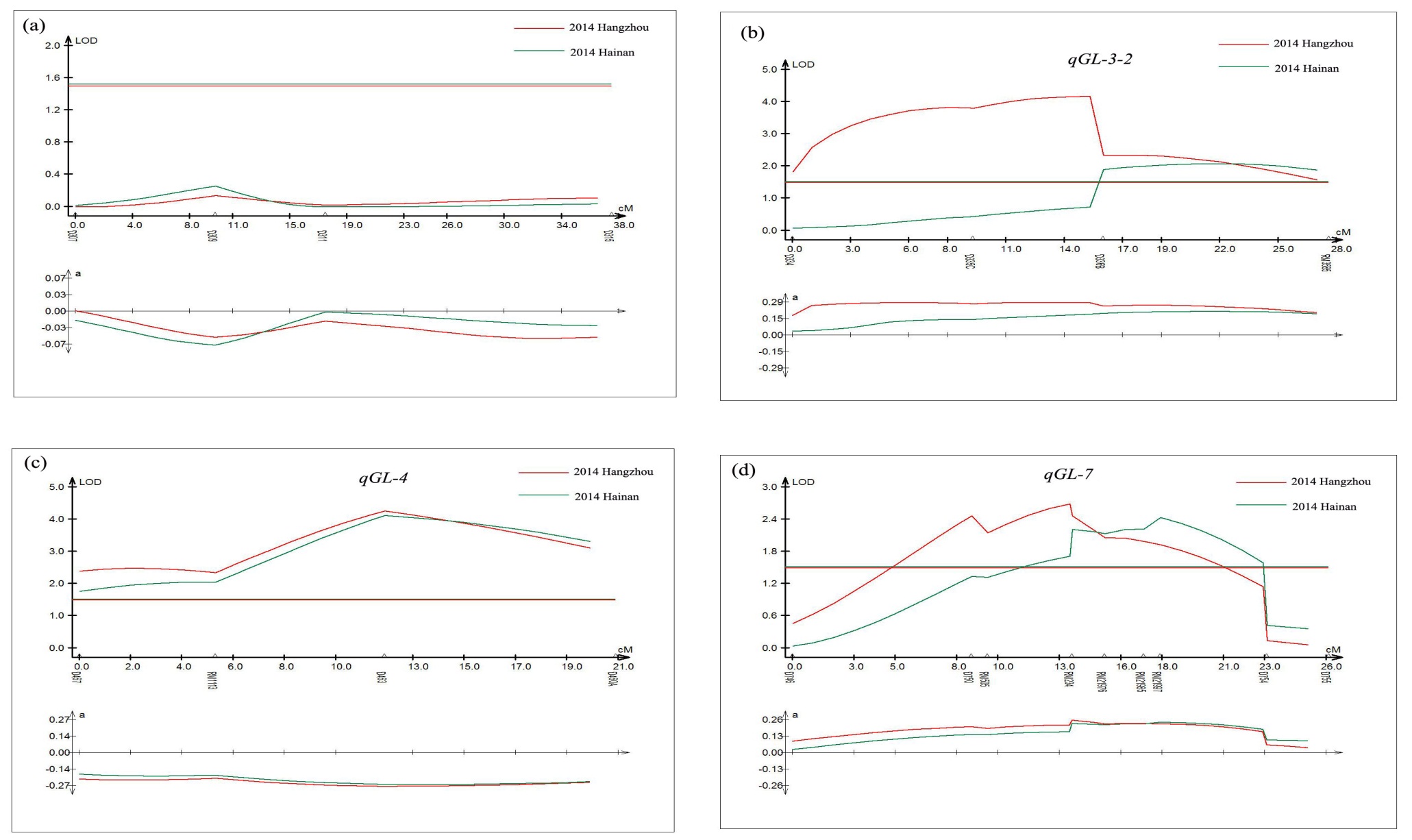


**S4 Fig.** **LOD score curves of quantitative trait loci (QTLs) for grain length detected in an F_7_ recombinant inbred line (RIL) and an F_8_ RIL mapping population derived from the cross between the *japonica* variety ‘Lemont’ and the *indica* variety ‘Yangdao 4’ and grown in 2014 in Hangzhou and Hainan, respectively, using composite interval mapping.** LOD: logarithm of odds; a: Additive effect. Additive effect is the effect of substituting a Yangdao 4 allele for a Lemont allele. Additive effect values are presented in Table 1.
