# Supplementary material for: Combination of Eight Alleles at Four Quantitative Trait Loci Determines Grain Length in Rice
Source: PLoS One. 2016 Mar 4;11(3):e0150832. doi: 10.1371/journal.pone.0150832 (PMC4778864; doi:10.1371/journal.pone.0150832)
Supplement: S5 Fig — (DOCX) [file pone.0150832.s005.docx]

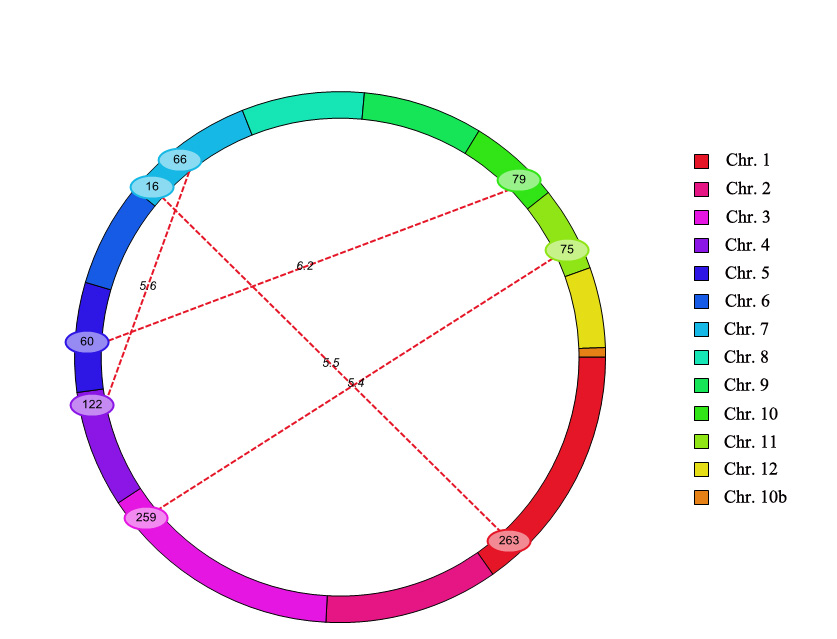


**S5 Fig.** **Digenic epistatic loci detected in an F_2_ mapping population derived from the cross between the *japonica* variety ‘Lemont’ and the *indica* variety ‘Yangdao 4’ and grown in 2011 in Hangzhou, using inclusive composite interval mapping.** Numbers on the dotted lines indicate the LOD scores. Numbers within the ellipses indicate the position (cM) of the digenic epistatic loci. Markers used for the genotyping of each chromosome are presented by Wen et al. [32].
