# Supplementary material for: Combination of Eight Alleles at Four Quantitative Trait Loci Determines Grain Length in Rice
Source: PLoS One. 2016 Mar 4;11(3):e0150832. doi: 10.1371/journal.pone.0150832 (PMC4778864; doi:10.1371/journal.pone.0150832)
Supplement: S1 Table — (DOCX) [file pone.0150832.s006.docx]

**S1 Table.** **Markers used to construct a linkage map for detecting quantitative trait loci (QTLs) in an F_2_ population derived from the cross between the *japonica* variety ‘Lemont’ and the *indica* variety ‘Yangdao 4’ and grown in 2012 in Hangzhou.**

| Chr. | Chr. 1 | | Chr. 3a | | Chr. 3b | | Chr. 4 | | Chr. 6 | | Chr. 7a | | Chr. 7b | | Chr. 8 | | Chr. 10 | | Chr. 11 | | Chr. 12 | |
| --- | --- | --- | --- | --- | --- | --- | --- | --- | --- | --- | --- | --- | --- | --- | --- | --- | --- | --- | --- | --- | --- | --- |
| Marker name and marker position (cM) | D133B | 0 | D307 | 0 | RM3585 | 0 | D456 | 0 | D653 | 0 | D701 | 0 | D745 | 0 | D856 | 0 | D1053 | 0 | D1103 | 0 | D1260 | 0 |
|  | D134B | 7.1 | D309 | 13.9 | D336B | 10.4 | D463 | 21.2 | RM1340 | 16.5 | RM3831 | 8 | D750 | 19.4 | D860 | 12.4 | RM496 | 2.7 | RM26155 | 28.7 | D1252 | 8 |
|  | D140A | 43.4 | D311 | 22.6 | D335C | 17 | RM1113 | 29.7 |  |  | D705 | 18.5 | RM505 | 21.9 |  |  | D1048 | 16.4 | D1113 | 43.3 | RM1246 | 12 |
|  | D144A | 73.5 |  |  | D334 | 27.9 | D468 | 36 |  |  |  |  | RM234 | 27.8 |  |  | D1042 | 50.4 |  |  | D1239 | 19.4 |
|  |  |  |  |  | D333B | 31.4 |  |  |  |  |  |  | D755 | 35.5 |  |  |  |  |  |  |  |  |
|  |  |  |  |  | D331B | 41 |  |  |  |  |  |  | D760 | 62.5 |  |  |  |  |  |  |  |  |
|  |  |  |  |  | D328B | 60.7 |  |  |  |  |  |  | RM248 | 64.4 |  |  |  |  |  |  |  |  |
|  |  |  |  |  | D325A | 86.3 |  |  |  |  |  |  |  |  |  |  |  |  |  |  |  |  |
