# Supplementary material for: Combination of Eight Alleles at Four Quantitative Trait Loci Determines Grain Length in Rice
Source: PLoS One. 2016 Mar 4;11(3):e0150832. doi: 10.1371/journal.pone.0150832 (PMC4778864; doi:10.1371/journal.pone.0150832)
Supplement: S2 Table — (DOCX) [file pone.0150832.s007.docx]

**S2 Table.** **Pairs of digenic epistatic loci detected in an F_2_ population derived from the cross between the *japonica* variety ‘Lemont’ and the *indica* variety ‘Yangdao 4’ and grown in 2011 in Hangzhou, using inclusive composite interval mapping.**

| Chr. 1^a^ | Left Marker 1 | Right Marker 1 | Chr. 2^b^ | Left Marker 2 | Right Marker 2 | LOD^c^ | PVE(%)^d^ | Add1^e^ | Add2^f^ | Dom1^g^ | Dom2^h^ | Add by Add^i^ | Add by Dom^j^ | Dom by Add^k^ | Dom by Dom^l^ |
| --- | --- | --- | --- | --- | --- | --- | --- | --- | --- | --- | --- | --- | --- | --- | --- |
| 1 | D133B | D134B | 7 | D701 | RM3831 | 5.5 | 13.6 | -0.06 | 0.23 | -0.17 | -0.21 | 0.03 | 0.02 | -0.21 | 0.42 |
| 4 | D456 | D463 | 7 | D709 | RM6574 | 5.6 | 27.2 | -0.33 | -0.14 | 0.12 | 0.23 | 0.08 | 0.18 | 0.22 | -0.34 |
| 5 | D516 | D531 | 10 | D1038 | D1042 | 6.2 | 12.4 | 0.14 | -0.10 | -0.20 | -0.07 | 0.11 | -0.32 | 0.08 | 0.22 |
| 3 | RM5813 | D333B | 11 | D1133 | D1142 | 5.4 | 13.9 | -0.04 | -0.02 | 0.26 | 0.12 | 0.14 | 0.14 | -0.08 | -0.28 |

^a^Chr.1: first chromosome position of detected digenic epistatic loci.

^b^Chr.2: second chromosome position of detected digenic epistatic loci.

^c^LOD: logarithm of odds.

^d^PVE (%): phenotypic variation explained by digenic epistatic interaction.

^e^Add1: estimated additive effect of the first digenic locus.

^f^Add2: estimated additive effect of the second digenic locus.

^g^Dom1: estimated dominance effect of the first digenic locus.

^h^Dom2: estimated dominance effect of the second digenic locus.

^i^Add by Add: additive-by-additive digenic interaction.

^j^Add by Dom: additive-by-dominance digenic interaction.

^k^Dom by Add: dominance-by-additive digenic interaction.

^l^Dom by Dom: dominance-by-dominance digenic interaction.
