# Supplementary material for: Combination of Eight Alleles at Four Quantitative Trait Loci Determines Grain Length in Rice
Source: PLoS One. 2016 Mar 4;11(3):e0150832. doi: 10.1371/journal.pone.0150832 (PMC4778864; doi:10.1371/journal.pone.0150832)
Supplement: S3 Table — (DOCX) [file pone.0150832.s008.docx]

**S3 Table.** **Two-way analysis of variance used to confirm the digenic epistatic loci detected in an F_2_ population derived from the cross between the *japonica* variety ‘Lemont’ and the *indica* variety ‘Yangdao 4’ and grown in 2011 in Hangzhou, using inclusive composite interval mapping.**

|  | DF | Type Ⅰ SS | Mean square | F value | *P* |
| --- | --- | --- | --- | --- | --- |
| D133B | 2 | 0.51 | 0.26 | 1.47 | 0.23 |
| D701 | 2 | 0.84 | 0.42 | 2.39 | 0.09 |
| D133B × D701 | 4 | 2.32 | 0.58 | 3.32 | 0.01* |
| D463 | 2 | 4.49 | 2.24 | 14.68 | <0.01 |
| D709 | 2 | 1.67 | 0.83 | 5.45 | 0.01 |
| D463 × D709 | 4 | 1.73 | 0.43 | 2.82 | 0.03* |
| D516 | 2 | 1.92 | 0.96 | 5.83 | <0.01 |
| D1038 | 2 | 0.59 | 0.29 | 1.79 | 0.17 |
| D516 × D1038 | 4 | 2.35 | 0.59 | 3.57 | <0.01** |
| D333B | 2 | 1.01 | 0.5 | 3.07 | <0.05 |
| D1133 | 2 | 0.13 | 0.06 | 0.38 | 0.68 |
| D333B × D1133 | 4 | 2.19 | 0.55 | 3.33 | 0.01* |

DF: degrees of freedom; SS: sum of squares

*, *P* < 0.05; **, *P* < 0.01.
